# Increased Urine IgM and IgG_2_ Levels, Indicating Decreased Glomerular Size Selectivity, Are Not Affected by Dalteparin Therapy in Patients with Type 2 Diabetes

**DOI:** 10.1155/2012/480529

**Published:** 2012-02-12

**Authors:** Ole Torffvit, Majid Kalani, Jan Apelqvist, Björn Eliasson, Jan W. Eriksson, Kerstin Brismar, Gun Jörneskog

**Affiliations:** ^1^Department of Nephrology, Institution of Clinical Sciences, Lund University Hospital, 22185 Lund, Sweden; ^2^Primary Care Unit, Capio-Citykliniken, Björkhemsvägen 15C, 29154 Kristianstad, Sweden; ^3^Department of Cardiology, Danderyd Hospital, 18288 Stockholm, Sweden; ^4^Department of Endocrinology, Malmö University Hospital, 20502 Malmö, Sweden; ^5^Diabetes Centrum, Sahlgrenska University Hospital, 41345 Göteborg, Sweden; ^6^Department of Molecular and Clinical Medicine, Sahlgrenska University Hospital, 41345 Göteborg, Sweden; ^7^Department of Endocrinology, Karolinska University Hospital, 17164 Solna, Sweden; ^8^Department of Clinical Sciences, Danderyd Hospital, Karolinska Institutet, 18288 Stockholm, Sweden

## Abstract

Fifty-four type 2 diabetic patients with neuroischemic foot ulcers were randomised to treatment with 5000 IU of dalteparin, (*n* = 28), or physiological saline, (*n* = 26), once daily until ulcer healing or for a maximum of 6 months. Thirty-three patients had normo-, 15 micro-, and 6 macroalbuminuria. The urinary levels of IgM and IgG_2_ were elevated in 47 and 50 patients, respectively. Elevated urinary levels of IgM and IgG_2_ indicate decreased glomerular size selectivity. Urine IgM levels were associated with IGF-1/IGFBP-1 and IGFBP-1 levels. Dalteparin treatment increased urinary levels of glycosaminoglycans (*P* < 0.001) and serum IGFBP-1 (*P* < 0.05) while no significant effects were seen in any of the other studied parameters. In conclusion, dalteparin therapy in patients with type 2 diabetes had no effects on urinary levels of albumin, IgM, or IgG_2_ despite significantly increased glycosaminoglycans in urine. Elevated urinary levels of IgM and IgG_2_ might be more sensitive markers of renal disease than albuminuria in patients with type 2 diabetes and antihypertensive therapy.

## 1. Introduction

Albuminuria is a marker of diabetic nephropathy and a strong predictor of widespread vascular damage [1]. The Steno hypothesis held that genetically based disturbances in the production or sulphation of heparan sulphate (HS) lead to a reduction of sulphated and negatively charged HS glycosaminoglycan (GAG) side chains. Negatively charged HS GAG side chains are normally found in the extracellular matrix and vascular basement membranes. High blood glucose levels lead to lower activity of the enzymes involved in GAG metabolism and sulphation of HS [2]. A reduction of negatively charged HS GAG may induce an increased transvascular permeability of negatively charged plasma proteins, which promotes vascular and glomerular changes [1, 3–5]. Positive effects of heparin on diabetic nephropathy have been shown in experimental studies [6–8]. In humans with diabetes, several studies have shown a reduction of urinary albumin excretion during treatment with unfractioned heparin, low-molecular-weight heparins (LMWH), or oral treatment with sulodexide, suggesting that these compounds can improve GAG metabolism and sulphation of HS [3]. Thus, in patients with type 1 diabetes, treatment with unfractionated heparin, sulodexide or LMWH decreased the albumin excretion rate [9, 10], whereas in type 2 diabetes, the effect on albuminuria seems less consistent [3]. In a study by Nielsen et al., three weeks of daily injections of the LMWH tinzaparin had no effect on albuminuria in patients with type 2 diabetes [11]. We have earlier reported an improved outcome of chronic neuroischemic foot ulcers in patients with diabetes during long-term treatment with dalteparin [12]. The beneficial effects of dalteparin on ulcer outcome involved an inhibitory effect on thrombin generation and improved haemostatic and microvascular functions [13]. The described effects of dalteparin may be beneficial not only for outcome of neuroischemic diabetic foot ulcers but also for other complications, such as diabetic nephropathy. Thus, the aim of this ancillary study was to investigate the effect of treatment with the LMWH dalteparin on proteinuria in patients with diabetes and severe vascular complications. The selectivity of the glomerular filter was studied by analyzing the urinary excretion of molecules of different size and charges [14–17], that is, IgM was analysed for determination of the size, and IgG_2_ and IgG_4_ for determination of the neutral and negative charges of the glomerular filter, respectively. The glomerular mesangial matrix turnover was assessed by measuring the urinary excretion of cytokine transforming growth factor beta 1 (TGF*β*1) [18]. Furthermore, we analyzed insulin-like growth factor 1 (IGF-1) and IGF-binding protein 1 (IGFBP-1) since the IGFBP-1 [19] and IGF1 have been shown to be associated with diabetes nephropathy independent of the degree of albumin [20]. It has been speculated that low IGF-1 activity may induce apoptosis or loss of podocytes and thus lead to glomerulosclerosis [21].

## 2. Subjects and Methods

### 2.1. Subjects

Of the previously described 87 diabetic patients [12] with peripheral arterial occlusive disease (PAOD) and chronic foot ulcer, 54 type 2 diabetic patients who completed the urine collections were included in the present study. All patients were treated with 75 mg aspirin once daily since at least four weeks before randomization and throughout the study period.

### 2.2. Methods

Prospective, double-blind, and placebo-controlled multicenter study to evaluate the effects of dalteparin (Fragmin, Pfizer) primarily on healing of neuroischemic foot ulcers [12] and secondarily on haemostatic and microvascular functions [13], and renal excretion of proteins. The patients were randomized to treatment with 0.2 mL daily subcutaneous injections of dalteparin (25000 U/mL) or physiological saline until ulcer healing or for a maximum of six months.

Timed urine collections from three consecutive nights before and at the end of treatment were stored at −20°C and analyzed at the Renal Laboratory, Lund. Microalbuminuria was defined as a mean value of the urine collections of 20 to 200 *μ*g/min or u-albumin/creatinine ratio of 3–30 mg/mmol. An excretion below these levels was defined as normo- and an excretion above as macroalbuminuria.

Urine albumin [22], total GAG [23], IgM [24], IgG_2_, and IgG_4_ [25] were analyzed as previously described. Biologically active TGF*β*1 was analyzed with a commercially available assay (Emax Immunoassay System, Promega Corp., Madison, WI, USA). U-creatinine was analyzed with an enzymatic method (EKTACHEM, Clinical Chemistry Slide, Johnson & Johnson Clinical Diagnostics, Rochester, NY, USA). HbA_1c_ was analyzed by an immunoturbidimetric method (UNIMATE 3 HbA_1c_, Roche Diagnostics). HsCRP and S-AA were measured using particle-enhanced immunonephelometric methods (BN, Dade Behring). IGF-I [26] and IGFBP-1 [27] were determined in serum by radioimmunoassays (RIAs).

### 2.3. Statistical Methods

Data are shown as mean and SD and skewed variables as median (minimum and maximum values). For differences within subjects we used Friedman's test, with Wilcoxon signed-rank test as post hoc test. The chi-square test was used to compare differences in the distribution of categorical variables. For testing of differences between subject groups, the Mann-Whitney *U* test was used. *P* values below 0.05 were considered significant (2-tailed). The statistical program SPSS was used.

### 2.4. Ethical Considerations

The study protocol was approved by the local ethics committee of each centre and the Swedish Medical Products Agency. Written informed consent was obtained from all patients.

## 3. Results

### 3.1. Patient Characteristics

Fifty-four patients with type 2 diabetes were able to leave timed urine collections from three consecutive nights before and at the end of treatment period. All patients had PAOD, peripheral neuropathy, and chronic foot ulcers. Seven patients in the dalteparin and 10 in the placebo group had suffered from myocardial infarction, and two patients in the placebo group had undergone leg amputation. Except for more ex-smokers in the placebo group, the baseline patient characteristics were not different between the two groups (Table 1). Levels of HbA_1c_ at baseline (Table 1) and at the end of treatment period (dalteparin: 7.0 (4.9–10.8)%; placebo: 6.3 (4.6–8.7)%) were not significantly different between the groups. Ten patients in the dalteparin group and 11 in the placebo group had micro- or macroalbuminuria (Table 1). Thirty-six patients, including 23 patients with normoalbuminuria, were on antihypertensive treatment (Table 1).

### 3.2. Treatment Period

The treatment period with dalteparin was not significantly different from the treatment period in the placebo group. It lasted for median 26 and range 8 to 26 weeks.

### 3.3. Renal Parameters

**Figure 1:**
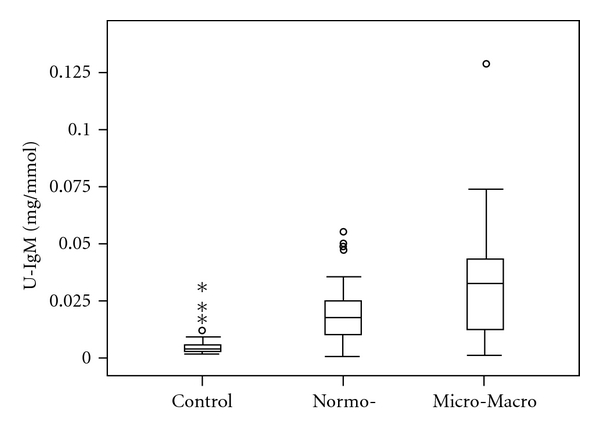
Box-plot of levels of IgM in control subjects compared with patients with normo- and micro- or macroalbuminuria at baseline (*P* < 0.001). Normo- versus micro- or macroalbuminuria (*P* = 0.05).

At baseline, 33 patients had normo-, 15 micro-, and 6 macroalbuminuria. Thirty-six patients, including 23 patients with normoalbuminuria, were on antihypertensive treatment (Table 1). Ten patients in the dalteparin group and 11 in the placebo group had micro- or macroalbuminuria (Table 1). Forty-seven patients showed elevated urinary levels of IgM (Figure 1), while 50 patients had elevated urinary levels of IgG_2_, both indicating decreased glomerular size selectivity. Twelve patients had a ratio of IgG_2_/IgG_4_ less than 1 indicating decreased charge selectivity, while 8 patients had urine levels of GAG less than or equal to 2 mg/mmol.

 Urinary GAG increased from 2.43 (0–8.65) mg/mmol at baseline to 3.40 (1.25–8.0) mg/mmol during dalteparin therapy (*P* < 0.001), while GAG levels were not significantly changed in the placebo group (baseline: 2.53 (0–8.99) mg/mmol). All other urinary parameters, including glomerular filtration rate (GFR), were not significantly different between dalteparin- and placebo-treated patients at baseline or at the end of treatment (Tables 1 and 2; data at end of treatment not shown). Baseline levels of systolic blood pressure, HbA_1c_, S-creatinine, S-HsCRP, S-AA, S-IGF, S-IGFBP-1, U-GAG, and U-IgG_2_/IgG_4_ were not significantly different between patients with normo- and micro- or macroalbuminuria (Table 2), and no associations were found between urinary GAG, HbA_1c_, and blood pressure levels and the urinary parameters. Urine levels of IgG_2_ and IgG_4_ were higher in patients with micro- or macroalbuminuria than in those with normoalbuminuria (*P* < 0.05) (Table 2). The dalteparin-induced increase in urinary GAG was independent of the degree of albuminuria, and no gender differences were found (data not shown). No significant effects of dalteparin treatment were seen on the urinary excretion of proteins in either patients with normoalbuminuria, or in patients with micro- or macroalbuminuria (Tables 3 and 4).

### 3.4. Comparisons with Data from Control Subjects

In comparison with control subjects [28], the urinary levels of IgG_2_ were higher in the patients with micro- or macroalbuminuria while normal in those with normoalbuminuria. Levels of IgG_4_ were normal, while IgG_2_/IgG_4_ ratios, and IgM and TGF*β*1-values [18] were increased irrespective of the level of albuminuria (for reference values, see Table 2).

### 3.5. Inflammatory Parameters, IGF-1 and IGFBP-1

The levels of hsCRP, SAA, S-IGF-1, and S-IGFBP-1 were similar in the dalteparin and placebo groups at baseline and during the treatment period (data not shown), except for S-IGFBP-1 which increased in patients with micro-macroalbuminuria in comparison with placebo-treated patients (Tables 3 and 4). No associations were found with any of the urinary parameters or HbA1c levels. S-IGF-1 was negatively associated with systolic BP at entry (*r* = −0.304, *P* = 0.048, *n* = 43). SAA and hsCRP were negatively associated with systolic BP at endpoint (*r* = −0.294, *P* = 0.038, *n* = 50 and *r* = −0.292, *P* = 0.036, *n* = 52; resp.). No differences were found between normo- and micro- or macroalbuminuric patients (Table 2). However, urine IgM/creatinine ratio was correlated to IGF1/IGFBP1 (*r* = −0.36, *P* = 0.008, *n* = 54) and IGFBP1 (*r* = 0.34, *P* = 0.013, *n* = 54).

## 4. Discussion

The results of the present study show that six months of treatment with the LMWH dalteparin had no effect on glomerular function, inflammatory parameters, or urinary levels of proteins despite an increased urinary excretion of GAG. Our results extend the findings of an earlier study showing that three weeks of LMWH treatment had no effect on albuminuria in patients with type 2 diabetes [11]. These findings are in contrast to the effect seen in type 1 diabetic patients showing a reduced albuminuria during one-to-three month treatment with either unfractionated heparin or LMWH [9, 10]. The reason for this discrepancy in effects of heparins on urinary excretion of proteins between patients with type 1 and type 2 diabetes is unclear and cannot be explained by the present study. However, the structure of the heparin molecule might be of importance since mixed compositions of sulphated GAG and heparan sulphate, for example, danaparoid [31], seemed to be more effective in type 2 diabetic patients. Another compound sulodexide which is a mixture of glucuronyl glycos aminoglycan and dermatan sulphate in an early study seemed to be effective [32], while a later double-blind randomized study showed that the drug was unable to decrease urine albumin excretion in patients with type 2 diabetic nephropathy and microalbuminuria [33].

The levels of total urinary GAG increased during treatment with dalteparin, which may be due to restitution of glomerular GAG or simply by an increased urinary excretion of dalteparin [34]. The low-molecular-weight heparin dalteparin is composed of strongly acidic sulphated polysaccharide chains with an average molecular weight of 5000 and about 90% of the material within the range 2000–9000. An earlier study by our group showed normal excretion of GAG in normoalbuminuric type 1 diabetic patients, while the levels were decreased in micro- and macroalbuminuric patients [25]. In contrast, the present study showed no significant differences in the levels of GAG in patients with normal or increased urinary excretion of albumin.

Normally, the urinary levels of IgG_2_, IgG_4_, and IgM are undetectable. In the present study, more patients had increased levels of urinary IgG_2,_ IgG_2_/IgG_4_ ratio, or IgM than patients who had micro- or macroalbuminuria. The loss of negative charges of the glomerular capillary wall causes the “effective” small pore radius vis-à-vis negatively charged macromolecules to increase to ~4.5 nm, which allows the passage of albumin. Larger proteins, such as IgG (mol radius 5.5 nm) or IgM (mol radius 12 nm), are still unable to pass across this pathway. IgG passes the glomerular capillary walls through the large pores, while IgM can permeate the glomerular capillary wall solely through the shunts [28]. Thus, increased transport of IgG indicates increased density of large pores, while increased concentration of urine IgM indicates increased density of shunts in the glomerular capillary wall [28]. In the present study, 47 patients had detectable levels of IgM, while only 21 patients had albuminuria; thus, renal disease was found in patients not detected by analysis for urine albumin. Since peripheral arterial occlusive disease is a marker of widespread vascular disease, one could expect that more patients in the present study would have had albuminuria. One reason for the low number may be antihypertensive medication, which was common in the present study. Thus, albuminuria may be a less sensitive parameter for evaluating nephropathy in patients on antihypertensive treatment. LMWH had no effect on the other urinary parameters studied, that is, IgG_2_/IgG_4_, IgG_2_ or IgM. In recent years new technologies of genomic analysis and proteomic approaches have detected several new markers for renal disease like neutrophil gelatinase-associated lipocalin (NGAL), kidney injury molecule-1 (KIM-1), and podocin [35–37]. However, the substances have not been proved to be of significant prognostic value and thus the findings have not resulted in improvement of the management of diabetic nephropathy [38, 39].

We have previously found higher renal excretion of IgM, IgG_2_, and IgG_2_/IgG_4_ in type 2 than in type 1 diabetic patients with overt nephropathy despite similar degree of albuminuria [28]. Thus, proteinuria in type 2 diabetic patients may be caused by an alteration of the size selective properties of the glomerular capillary wall, including the occurrence of nondiscriminatory “shunt pathways,” rather than by charge selectivity [15]. We have previously found increased excretion of IgM to be a poor prognostic factor [40]. The IGFBP-1 gene has been suspected to be protective for nephropathy [19], possibly through altered IGFBP-1 binding to IGF-1 with local effect in the kidney. In the present study in patients with vascular disease we found increased excretion of IgM, and thus these patients may be at increased risk. We furthermore found a positive association between IGFBP-1 and excretion of IgM indicating that high IGFBP-1 may be associated with glomerular damage. Thus, we were able to confirm decreased levels of IGF-1 and increased levels of IGFBP-1 in type 2 diabetes patients with nephropathy [20]. Furthermore, IGFBP-1 increased to significantly higher levels in patients treated with dalteparin than in placebo-treated ones. The reason for these increased levels is not known but may be due to reduced proteolysis of IGFBP-1. In line with a study by Sharma et al. [18], the present study showed increased urinary levels of TGF*β*1 in patients with type 2 diabetes. However, the levels of TGF*β*1 were also unaffected by dalteparin treatment.

In conclusion, the present study showed no effects of dalteparin on the glomerular filter despite increased S-IGFBP-1 levels and urinary levels of GAG. Thus, the study indicates that proteinuria in type 2 diabetic patients may be caused by an alteration of the size-selective properties of the glomerular capillary wall. IgM and IgG_2_ seem to be better markers than albuminuria for severe vascular disease.

## Figures and Tables

**Table 1:** Baseline characteristics of 54 patients randomized to dalteparin or placebo.

	All	Dalteparin	Placebo
	*N* = 54	*N* = 28	*N* = 26
Age (years)	75 (54–90)	73 (57–86)	75 (54–90)
Gender (male/female)	37/17	17/11	20/6
Smoker/ex-smoker/nonsmoker (*n*)	9/14/31	4/3/21*	5/11/10
HbA_1c_ (%)	6.7 (5.0–11.0)	6.9 (5.1–11)	6.9 (5.0–9.6)
Diabetes duration (years)	17 ± 9	17 ± 10	16 ± 8
Tablets/insulin/tablets + insulin/diet (*n*)	10/31/8/5	4/18/3/3	6/13/5/2
Antihypertensive treatment (*n*) (ACE/*β*/Ca/diuretic/other)	10/13/5/22/4	5/6/3/14/0	5/7/2/8/4
Systolic blood pressure (mmHg)	158 ± 22	160 ± 22	155 ± 22
Diastolic blood pressure (mmHg)	80 ± 11	78 ± 9	82 ± 12
P-Creatinine (*μ*mol/L)	83 (53–160)	83 (57–130)	84 (53–160)
GFR (mL/min)	74 (17–218)	74 (34–190) (*n* = 22)	80 (17–218) (*n* = 22)
*Albuminuria (normo/micro/macro): *			
Baseline (*n*)	33/15/6	18/8/2	15/7/4
At endpoint (*n*)	35/12/7	18/7/3	17/5/4

*P < 0.05 versus placebo. Data are given as mean ± SD, or as median and minimum-maximum values. GFR: glomerular filtration rate: creatinine clearance.

**Table 2:** Baseline values in patients grouped with normo- or micro- and macroalbuminuria.

	Normoalbuminuria	Micro- and macroalbuminuria
	*N* = 33	*N* = 21
Age (years)	74 (54–90)	75 (61–86)
Diabetes duration (years)	15 ± 9	19 ± 8
Gender (male/female)	21/12	16/5
Systolic blood pressure (mmHg)	150 (115–210)	160 (135–215)
Diastolic blood pressure (mmHg)	80 (60–100)	85 (60–105)
S-HbA_1c_ (%)	6.5 (5.0–9.9)	6.9 (5.1–11.0)
S-Creatinine (*μ*mol/L)	81 (53–160)	85 (65–128)
S-Hs CRP (mg/L)	9.4 (0.9–118)	2.7 (0.3–78.2)
S-AA (mg/L)	5.5 (1.2–415)	5.1 (1.7–127)
S-IGF-1 (*μ*g/L)	134 (47–384)	115 (49–269)
S-IGFBP-1 (*μ*g/L)	41 (15–310)	60 (8–313)
U-Glycosaminoglycan (mg/mmol)^a^	2.7 (0–8.7)	2.6 (0–11.1)
U-IgG_2_ (mg/mmol)^b^	0.18 (0–8.1)	0.85 (0–99)*
U-IgG_4_ (mg/mmol)^c^	0.06 (0–7.7)	0.27 (0–28.7)*
U-IgG_2_/IgG_4_^d^	3.1 (0.04–31.0)	3.3 (0.76–10.5)
U-IgM (mg/mmol)^e^	0.02 (0–0.06)	0.03 (0–0.13)
U-TGF*β*1 (ng/mmol)	3.2 (1.1–379)	4.5 (1.4–16.5)

*P < 0.05 versus normoalbuminuria. Data are given as median and range (min-max). Urine data are the ratio between urine protein and urine creatinine. ^a^Reference values for U-GAG: 2.9 (2.0–4.4) mg/mmol [25]; ^b^U-IgG_2_: 0.19 ± 0.14 mg/mmol; ^c^U-IgG_4_: 0.35 ± 0.25 mg/mmol; ^d^U-IgG_2_/IgG_4_: 2.3 ± 0.7; ^e^U-IgM: 0.002 ± 0.001 mg/mmol [28]. IGFBP-1 (15–45) [29, 30].

**Table 3:** Diabetic patients with normoalbuminuria: effects of treatment on urinary indices.

	Dalteparin		Placebo	
	Baseline	At endpoint	Baseline	At endpoint
	*n* = 18	*n* = 18	*n* = 15	*n* = 15
U-Albumin (mg/mmol)	0.81 (0.07–2.39)	0.77 (0.06–4.97)	0.81 (0.13–4.09)	0.80 (0.19–6.73)
U-IgG_2_ (mg/mmol)	0.19 (0–8.14)	0.14 (0–7.79)	0.18 (0.06–2.64)	0.16 (0.01–3.79)
U-IgG_4_ (mg/mmol)	0.05 (0–7.68)	0.04 (0–1.08)	0.07 (0.02–2.51)	0.06 (0.02–4.63)
U-IgG_2_/u-IgG_4_	3.49 (0.04–31)	2.20 (0.37–44.59)	2.38 (0.41–8.58)	2.63 (0.54–18.7)
U-GAG (mg/mmol)	2.43 (0.86–8.65)	2.85 (1.32–8)^∗#^	3.32 (0–6.45)	2.53 (0.93–8.99)
U-IgM (mg/mmol)	0.02 (0–0.06)	0.02 (0–0.05)	0.02 (0–0.05)	0.02 (0–0.05)
TGF-*β*1 (mg/mmol)	3.2 (1.1–379)	5.17 (1.47–21.3)	3.4 (1.4–24.6)	3.32 (1.7–28.9)
GFR (mL/min)	70 (34–190)	65 (33–163)	99 (17–218)	86 (20–334)
	(*n* = 14)	(*n* = 13)	(*n* = 12)	(*n* = 12)
IGFBP-1 (*μ*g/L)	42 (21–310)	49 (27–315)	38 (15–98)	53 (15–97)

Data are given as the median (with minimum and maximum values in parentheses) of the ratio between urinary concentrations of substance and u-creatinine. *P < 0.05 versus placebo; ^#^ *P* < 0.05 versus baseline. GFR: glomerular filtration rate: creatinine clearance.

**Table 4:** Diabetic patients with micro- or macroalbuminuria: effects of treatment on urinary indices.

	Dalteparin		Placebo	
	Baseline	At end point	Baseline	At end point
	*n* = 10	*n* = 10	*n* = 11	*n* = 11
U-Albumin (mg/mmol)	8.5 (0.9–435)	11.3 (1.5–311)	23.2 (2.1–187)	7.9 (0.9–273) (*n* = 10)
U-IgG_2_ (mg/mmol)	0.46 (0–20.7)	0.83 (0.13–35.1)	2.99 (0.02–99.4)	2.60 (0–70.0)
U-IgG_4_ (mg/mmol)	0.21 (0–3.94)	1.02 (0.04–7.16)	0.50 (0.03–28.7)	0.15 (0–53.7) (*n* = 10)
U-IgG_2_/u-IgG_4 _	1.88 (1–10.5)	3.05 (0.33–9.16)	5.57 (0.8–9.7)	4.47 (0.35–24.1)
U-GAG (mg/mmol)	2.31 (0–4.52)	3.97 (1.25–6.1)^∗##^	2.70 (0–11.1)	2.49 (0–5.19) (*n* = 10)
U-IgM (mg/mmol)	0.03 (0–0.05)	0.03 (0.01–0.12)	0.03 (0–0.13)	0.02 (0–0.14)
TGF-*β*1 (mg/mmol)	4.44 (1.4–15.9)	4.09 (2.14–14.12)	4.5 (1.8–16.5)	3.5 (1.19–22.44) (*n* = 10)
GFR (mL/min)	74 (36–140) (*n* = 8)	76 (18–208) (*n* = 9)	71 (38–107) (*n* = 10)	67 (34–113) (*n* = 10)
IGFBP-1 (*μ*g/L)	66 (8–313)	105 (23–219)* (*n* = 9)	60 (20–130)	46 (10–161)

Data are given as the median (with minimum and maximum values in parentheses) of the ratio between urinary concentrations of substance and u-creatinine. *P < 0.05 versus placebo; ^##^ *P* < 0.01 versus baseline. GFR: glomerular filtration rate: creatinine clearance.

## Abbreviations

IgG: Immunoglobulin G
TGF*β*1: Transforming growth factor *β*1
IGF-1: Insulin-like growth factor 1
IGFBP-1: Insulin-like growth factor binding protein 1.
